# Measuring the Association Between Sports and Mental Health Among Mexican LGBTQ People: Psychological Distress Among Athletes, Spectators/Volunteers, and Non-Attendees During the 2023 Gay Games

**DOI:** 10.3390/ijerph22091353

**Published:** 2025-08-29

**Authors:** Casper H. Voyles, Alma Lilia Cruz Bañares, Carlos Ignacio Ahedo Rocha, Heleen Vermandere

**Affiliations:** 1 Department of Social Justice and Social Change, Hamline University, Saint Paul, MN 55104, USA; cvoyles01@hamline.edu; 2 Center for Evaluation Research and Surveys, National Institute of Public Health, Cuernavaca 62100, Mexico

**Keywords:** psychological distress, LGBTQ+, Gay Games, sports participation, Mexico

## Abstract

Mental health benefits from sport are widely established in the general population yet remain underexamined among LGBTQ individuals. Investigation of whether sports can promote LGBTQ mental well-being is warranted. The 2023 Gay Games in Guadalajara, Mexico, provided the opportunity to examine psychological distress among Mexican LGBTQ individuals with different involvement within the event (n = 111). Gay Games athletes (n = 32), spectators/volunteers (n = 41), and non-attendees (n = 38) completed an online questionnaire about mental health, self-reported health, experiences of discrimination, and fear during pride events. Among this sample, 18.8% of athletes reported any psychological distress (as measured by PHQ-4) compared to 36.6% of spectators/volunteers and 50.0% of non-attendees. Multivariable logistic regression was used to assess the correlation between psychological distress and Gay Games participation. The final adjusted models demonstrated that athletes (OR: 0.200; CI: 0.063, 0.630) and spectators/volunteers (OR: 0.310; CI: 0.097, 0.961) had lower odds of reporting psychological distress than non-attendees. Other significant potential confounders were being transgender (OR: 4.582; CI: 1.392, 15.071), having excellent/very good/good self-rated health (OR: 0.101; CI: 0.014, 0.708), and reporting fear during pride events (OR: 4.583; CI: 1.692, 12.401). These findings provide support for an inverse relationship between poor mental health and LGBTQ-inclusive sports participation and can inform future interventions to promote well-being for groups experiencing substantial mental health disparities.

## 1. Introduction

### 1.1. Overview

Disparities in sports participation reflect the social exclusion faced by lesbian, gay, bisexual, transgender, and queer (LGBTQ) communities more broadly. Indeed, sports are often considered to be a microcosm of greater society, a place where social tensions play out on the field in tandem with competition. Sexual and gender minority individuals often keep these identities discrete; gender minorities in particular face explicit exclusion, either due to a lack of inclusive teams and facilities, or because they are banned from sports altogether because of societal ideas about gender, fairness, and athletic ability.

Some sporting events, such as the Gay Games, challenge the sporting status quo by celebrating LGBTQ identities; the Gay Games have done this at an international level, occurring every four years since 1982. While the Gay Games have previously been studied from historical and social perspectives [1,2,3], to our knowledge, no published studies have examined the Gay Games explicitly through a health promotion lens. Examining the potential impact on health and well-being not only would enhance understanding of possible mechanisms to reduce health disparities experienced by the LGBTQ community but also honor the historical impetus for the event’s creation—to bring the community together as it faced the emerging HIV/AIDS epidemic in the early 1980s.

In the present day, the mental health of LGBTQ individuals around the world is impacted by stigmatization and discrimination of diverse sexual and gender identities and exacerbated by isolation from community. This is relevant within the context of sports as current public discourse about LGBTQ athletes creates tensions within and among institutions, athletes, and supporters at all levels of competition. While globally relevant, the relationships between mental health, sports, and LGBTQ identity have disproportionately been examined within the Global North. The 2023 Gay Games in Mexico was the first Latin American iteration of this event, which provided a unique opportunity to examine the potential impacts of inclusive sports participation within a region in which LGBTQ mental health disparities have been demonstrated.

This study aimed to begin to elucidate some of the unknown components of the relationship between LGBTQ mental health and sport within a Latin American context. These components consisted of what we considered to be possible causal mechanisms for this relationship; specifically, we considered possibilities that: (1) Gay Games athletic competition contributes to mental well-being; (2) an active lifestyle contributes to Gay Games participation; (3) overall mental well-being may contribute to Gay Games participation; and/or (4) Gay Games-related community and social benefits may contribute to mental well-being. We neither view these possibilities as mutually exclusive, nor expect that our results from this study can produce definitive clarity about this process. Rather, we aim to present results from this exploratory investigation with recommendations for further study; it is our expectation that additional clarity about this topic will provide rationale for strategies that maximize wellness for those who are underrepresented, excluded, or invalidated both in their sport.

### 1.2. Psychological Distress and LGBTQ Populations

Psychological distress, including depression, anxiety, and their related symptoms, is a public health issue faced by LGBTQ populations; for example, recent prevalence estimates of serious psychological distress in LGB people in the United States was 8.0% [4]. While more research is needed about mental health among LGBTQ people globally, evidence suggests that substantial psychological distress is a concern among LGBTQ people in Mexico, where samples have estimated the prevalence of suicidal ideation among LGBTQ people to be more than one-third [5]; these estimates are as high as 39% among sexual minorities and 58% among transgender people [6,7]. Both experiences of discrimination among LGBTQ people and HIV-related stigma have been found to be associated with poor mental health in Mexico [8,9], further suggesting the potential impact of multiple marginalization among the estimated 17.4% of men who have sex with men living with HIV in the country [10].

Some mental health disparities have been demonstrated among LGBTQ Mexicans of various gender identities and age groups. While sexual minority women in Mexico have a greater likelihood of psychological distress in the form of suicidal ideation [11] and panic disorders [12], Mexican gay men have been found to exhibit greater odds of depressive disorders [12]; Mexican youth and young adults, particularly males, are disproportionately affected [13]. In its 2024 report on the mental health of LGBTQ youth in Mexico, the Trevor Project found that half of youth surveyed ages 13–24 seriously considered suicide in the past year [14].

That between one-third and one-half of LGBTQ Mexicans experience significant psychological distress is a major cause of concern, suggesting that mental health promotion in this population could be beneficial. Physical activity supports mental health among LGBTQ people [15], and among people living with HIV in Mexico [8]. A potential avenue for intervention, therefore, may be inclusive spaces, such as the Gay Games, where people of diverse sexual orientations, gender identities, and expressions can obtain the benefits of physical activity.

However, a narrow view that assumes physical activity would be the only potential benefit of the Gay Games—or, that exercise in and of itself is sufficient to improve mental health—would fail to recognize the sociocultural factors and interpersonal connection that have an impact on well-being. The 2023 Gay Games event was framed by a specific interweaving of Mexican cultural norms, narratives, values, and structures (i.e., machismo culture, significant religious and family structures) that can affect LGBTQ mental health at a time where regional attention to this topic has been expanding [16].

### 1.3. Sports and Mental Health

Evidence increasingly suggests that sports participation is associated with positive mental health, perhaps in ways that extend beyond what general exercise can provide. Sports participation has been found to be negatively associated with depression among adolescents [17,18]. Similar findings have been extended to adults, as team sports participation is most strongly associated with mental health within the context of physical activity participation [19]. Similar trends between sports participation and improved mental health have been found in other parts of the world [20,21,22]. Quantitative evidence suggesting disparities in the benefits achieved from sport between subpopulations is concerning. US studies have indicated that, while protective against poor mental health outcomes for white adolescent boys, sports’ protective effect does not extend equally to those of other genders or races/ethnicities [23]. A study in Brazil also found that participation in team sports had a protective effect against mental health concerns for boys, but not for girls [24].

Whether the mental health benefits of sport are experienced among LGBTQ populations is a newer topic of inquiry. Scholars have directed more attention in the recent decade to the phenomenology of being LGBTQ within sport, as well as the mental health symptomology associated with such participation. Prevalence estimates of conditions such as anxiety or depression from epidemiological studies are hard to determine due to an underrepresentation of LGBTQ people in sports, yet evidence suggests tailored approaches may be needed to support this group’s mental well-being. Analysis of data from the National College Health Assessment highlights a disproportionate mental health burden among US college athletes identifying as sexual minorities [25], contradicting the common association between sports and positive mental well-being. One recent study suggests that among elite athletes, LGBTQ people experience greater psychological distress [26]; as elite and intercollegiate athletes are likely to experience substantially different stressors (and/or outcomes) related to sports participation, intentional examination of LGBTQ adult community sport and its connection to mental health is needed.

Within mainstream sports clubs, LGBTQ people’s participation in and of itself is likely not a sole indicator of positive mental health; rather, findings suggest that the social environment within athletic spaces—including camaraderie with, support from, and connection to teammates—is likely more influential in determining mental health of LGBTQ athletes [27]. A 2022 systematic review of articles relating to LGBTQ athletes showed concerning prevalence of poor mental health symptoms among these athletes, with negative aspects of the sporting environment (i.e., discrimination, homo/transphobia, bullying, and a concealment of one’s LGBTQ identity from other athletes) as possible contributors to such outcomes [28].

More localized studies have demonstrated that certain team dynamics are associated with lower likelihood of depression for LGBTQ youth athletes compared to LGBTQ youth who do not play such sports [29]. Such findings suggest that mental health benefits of team sports can be obtained by LGBTQ youth within some team context. However, fewer LGBTQ youth overall engage in team sports compared to their heterosexual and cisgender counterparts [29]. Evidence suggests that LGBTQ people may be more likely to drop out of team sports in the transition between adolescence and adulthood [30,31]. In part, the relative lack of participation and persistence within team sports settings may be attributed to fewer perceived benefits to physical and mental health as reported by this group [32].

Recent findings indeed demonstrate the associations between mental health and sports participation among LGBTQ athletes; however, at present, the scientific literature related to mental health and LGBTQ athletes within exclusively Latin American contexts remains limited. Anderson and Piedra [33] emphasize that existing scholarship on LGBTQ people’s experiences in sports spans swaths of both geography and time, making the relevance and applicability of findings from one temporally- and geographically located context to another inconsistent. In their example, just as research from American LGBTQ athletes in 2011 would not likely be valid in 2021, nor would those 2021 findings be as applicable to athletes in the Global South as they would be in Canada, the UK, or other nations considered within the Global North [33]. Therefore, the present study’s focus on the mental health of Mexican participants of an LGBTQ-oriented sporting event hosted within their country presents the opportunity to elucidate potential similarities and/or differences from other contexts.

### 1.4. The 2023 Gay Games

The 2023 Gay Games, co-hosted in Guadalajara, Mexico and Hong Kong, China, was the first iteration of this quadrennial event being held in either Latin America or in Asia. The addition of Guadalajara as co-host was touted as a testament to Mexico’s progress towards LGBTQ inclusion in recent years, particularly in the context of Latin America more broadly. Mexico’s sporting history includes some of the oldest LGBTQ sporting clubs in Latin America [34], and the relatively friendly political climate includes legal recognition and rights related to same-sex marriage, gender affirmation, and child adoption. However, broader norms related to masculinity, dominant religious beliefs, and the prevalence of anti-LGBTQ violence in Mexico suggest that LGBTQ inclusion has not been widely achieved, and the conservative institution of athletics may be a particularly difficult sector to shift. Indeed, some have pointed to the very limited queer visibility among Mexican athletes as one indicator of a lack of spaces for LGBTQ to participate openly in sports [35].

### 1.5. Theoretical Framework

Our conceptualization of this study arose within the context of the findings referenced above, as well as within a theoretical framework shaped by minority stress theory [36], as well as the rethinking of it as the social safety perspective [37]. Meyer’s influential 2003 work on minority stress articulated that health disparities faced by sexual minorities arose not because of LGBTQ identity itself, but rather the toll of cumulative stress from discrimination (and resultant psychological processes) experienced within a heteronormative and homophobic society [36].

In supplement to Meyer’s minority stress theory, the concept of social safety—the recognition of and belonging within a “protective social fabric” has been suggested as another contributor to health disparities among LGBTQ people [37]. Stigmatized people have much more limited access to social safety, and the resulting vigilance in social settings sustains physiological and psychological activity that wears the body and brain down over time [37,38] to an extent beyond those who are able to let their guard down in the presence of social support [37]. These two theories provide insight as to *how* certain spaces may contribute to LGBTQ mental health, and *why* differences in this relationship can be found between specific sites.

Scholarship within other settings (i.e., families, schools) elucidates more about these theorized processes. Studies within the realm of families of LGBTQ youth reveal that parental support is protective against mental health concerns like suicidality, depression, and distress [39,40]; in the absence of strong familial support, youth who find acceptance in other settings see their mental health improve [40]. Some of these non-family spaces—such as schools, neighborhoods, or broader communities with their varying policies and practices related to LGBTQ acceptance—create a social environment that is also associated with mental health for LGBTQ youth [41]. The association between accepting social space and mental health extends across the lifespan; the perception of LGBTQ acceptance in workplaces is associated with adult employee mental health [42], and religious community rejection is associated with poorer mental health about LGBTQ older adults [43].

Minority stress and social safety each continue to have relevance to the study of LGBTQ athletes, particularly as such scholarship extends geographically to areas around the world. Discrimination is indeed pervasive in many athletic environments. While these contexts are far from monolithic, sport overall is described as a “socially conservative institution” that is characterized by global homophobia and transphobia (p. 2, [33]). While published research on Mexican athletes’ attitudes about LGBTQ identities is limited, recent work on sexual orientation diversity demonstrates that discrimination against and discomfort about sexual minorities persist within Mexican sports [44]. Despite the overall lack of acceptance within sports, LGBTQ people do indeed seek out opportunities to compete. The “vetting” process through which LGBTQ young adults seek safe and accepting athletic opportunities, includes particular attention to cues such as mixed-gender competition and references to LGBTQ identity [45]. The Gay Games, with its explicit and highly visible focus on providing affirming athletic competition for sexual and gender minorities, is also a space for transnational social connection and support, opportunities which can shape experiences of one’s own identity and community connectedness [46]. Therefore, the 2023 iteration of the event in Guadalajara is an appropriate site for examining how participation in such a space correlates with actual mental health experiences.

### 1.6. The Current Study

The setting of the 2023 Gay Games in Mexico provided the opportunity for a multinational research team to examine the health profiles of Gay Games participants, spectators and volunteers, and non-attendees from the Mexican LGBTQ community and those traveling from abroad. The current analysis explored the associations between Gay Games participation and mental health among LGBTQ residents of Mexico. Specifically, we aimed to quantitatively assess the relationship of Gay Games participation and psychological distress while adjusting for potential confounders.

This study is intended to be just an initial step towards establishing causality within the relationship between Gay Games participation and mental health. Rather than assert causal claims, the goal of this investigation was to determine a correlation between psychological distress and Gay Games participation to provide initial insight related to the following possible causal scenarios and associated hypotheses for testing:

1. Participation in the Gay Games (especially as an athlete) contributes to mental health;
  **Hypothesis** **1.** *Gay Games athletes will report significantly lower odds of psychological distress compared to other groups [due to the unique benefits of Gay Games competition/participation];*
2. Because Gay Games athletes may receive mental health benefits gained from a physically active lifestyle prior to the event, reflecting lower distress during the event.
  **Hypothesis** **2.** *Athletes participating in the Gay Games will have lower odds of psychological distress compared to other groups [due to physical activity levels];*
3. Those who are in better mental health prior to the Gay Games will be more likely to attend (as athlete or spectator/volunteer), resulting in lower odds of distress for these groups;
  **Hypothesis** **3.** *People participating in the Gay Games as athletes or spectators/volunteers will have lower odds of psychological distress compared to other groups [due to self-selection];*
4. The LGBTQ-inclusive environment supports positive mental well-being for all attendees, including athletes, spectators, and volunteers;
  **Hypothesis** **4.** *Those who attend the Gay Games (as athlete or spectator/volunteer) will have lower odds of psychological distress compared to non-attendees [due to the presence of social safety at the Gay Games events].*

## 2. Methods

### 2.1. Study Design

This analysis was part of a larger mixed methods investigation consisting of a concurrent embedded study design in which a qualitative component complemented the quantitative component. For the present analysis, data collection consisted of a cross-sectional online survey during and immediately following the 2023 Gay Games. Prior to data collection, ethical approval was provided by Institutional Review Boards at both the National Institute of Public Health in Mexico and Hamline University, USA.

### 2.2. Participants

Eligibility Criteria. Persons eligible for the overall study met the following criteria: (1) identified as LGBTQ or other sexual or gender minority, (2) were located in Guadalajara (GDL, located in the state of Jalisco), Puerto Vallarta (PV, also in Jalisco), or Mexico City (CDMX) at the time of the Gay Games, (3) be at least 18 years old, (4) be able to complete a survey and interview in English or Spanish.

For the present analysis, we restricted inclusion to LGBTQ residents of Mexico who were at least 18 years old and completed at least 50% of the survey, in addition to the dependent variable (psychological distress) and primary independent variable of interest (participation type); this sample included those who participated in the Gay Games as an athlete or spectator/volunteer, as well as those who did not attend the event. Overall, 194 eligible respondents of all nationalities consented to participate, 142 of whom reported living in Mexico. Of those who answered at least 50% of the survey (n = 120), nine were excluded for missing data on one of the two key variables, yielding 111 for the final sample for analysis. All participants in the present analysis responded to the survey within 13 days of its launch at the midpoint of the event.

Recruitment. Participant recruitment was conducted via two primary mechanisms: (1) through outreach via Gay Games platforms throughout the ten days of the event, and (2) through partnerships with LGBTQ-friendly social service and/or sports organizations based in Mexico. These outreach strategies were supported by two research assistants familiar with the LGBTQ community in Mexico City (CDMX) and in Jalisco, and efforts were concentrated in GDL, PV, and CDMX. In-person outreach to spectators and athletes was conducted at Gay Games and other LGBTQ venues in Guadalajara throughout the ten days of the Gay Games event. Promotional material was displayed on social media of both the Gay Games-specific and broader LGBTQ-oriented partner organizations to enhance outreach to potentially eligible LGBTQ individuals who were not physically present at the sporting or related cultural events (i.e., non-attendees).

### 2.3. Data Collection

Overview. Survey data were collected electronically via the Qualtrics platform hosted by Hamline University. Informed consent was obtained electronically from participants after confirming that they had: (1) read the IRB-approved informed consent information presented visually the first screen and (2) downloaded the PDF version of the form provided via a link on the same page. Providing electronic consent allowed participants access to the survey to be taken on their own electronic devices Surveys took approximately 20 min to complete. Active recruitment occurred from the midpoint of the Gay Games (7 November 2023) for two weeks.

### 2.4. Survey Measures

The survey was developed by the research team in English, translated to Spanish, and then back-translated and assessed for cultural/linguistic appropriateness by bilingual members of the team. Participants had the option to complete an English or Spanish version of the survey.

Demographics. Demographic data included in bivariate analyses were as follows: age, transgender identity, country of residence, state of residence, educational attainment, income, and current LGBTQ association involvement. Educational attainment was dichotomized to any or no university education; income was dichotomized to below or above MXN$7000 biweekly; this cutoff point was determined as it was approximately double the income poverty line at the time of the survey (November 2023) [47]. Survey respondents were prompted to input their age in years. Our survey featured a drop-down list of Mexican states from which they could select their state of residence; during analysis, data were grouped into “Jalisco,” “Mexico City,” “(State of) Mexico,” and “Other.”

Gay Games Participation. Gay Games participation was asked as a “select all that apply” option that included: (1) as an athlete, (2) as a spectator of athletic events, (3) as an attendee of Gay Games cultural events, (4) as a volunteer, coach, or trainer, and (5) none of the above. All who participated as an athlete were categorized as Athletes, “None of the Above” as Non-Attendees, and those who attended Gay Games events as a non-athlete as Spectators/Volunteers. This grouping provides the opportunity to examine whether sports participation itself affected well-being independently from the community engagement from attending the event as a non-athlete.

*Psychological Distress.* Psychological distress was measured using the four-item version of the Patient Health Questionnaire (PHQ-4), which has been validated in English and Spanish [48,49,50]. The PHQ-4 measures the extent of depression and anxiety by asking “Over the last two weeks, how often have you been bothered by the following problems?” with a range of response options denoting frequency for the following concerns: “feeling nervous, anxious or on edge”; “not being able to stop or control worrying”; “feeling down, depressed, or hopeless”; and “little interest or pleasure in doing things.” Each of the four questions of the PHQ-4 are scored from 0–3, with overall scores on the PHQ-4 item ranging from 0–12. Higher scores correlate with increased distress; 0–2 indicates no distress, 3–5 indicates mild distress, 5–8 indicates moderate distress, and 9–12 indicates severe distress. We utilized the cutoff score of 3 or greater to indicate any amount of distress from mild to severe, in line with other sports scholars who have utilized it with LGBTQ samples [51].

Self-Rated Health. Self-rated health was measured via the following question: “How would you describe your overall physical health?” Response options included “Excellent,” “Very Good,” “Good,” “Fair,” “Poor,” and “Prefer not to say.” The responses were dichotomized with the first three options representing good health, and “Fair” and “Poor” representing poorer health.

Past Experiences of Discrimination. To assess past experiences of discrimination specific to LGBTQ people, we utilized the sexual behavior stigma scale validated in multiple contexts [52,53,54], including within Mexican populations [55]. The version validated in Mexican men who have sex with men (MSM) is a 13-item scale consisting of three subscales related to public discrimination (eight items), healthcare discrimination (two items), and discrimination from family and friends (three items). Responses for each survey item were dichotomized and averaged for each subscale, resulting in scores ranging from 0.0–1.0 for each subscale; a score of 1.0 indicates affirmative responses to each item about experiences of discrimination within each domain.

Fear of Pride Events. Because of the framing of our study in line with minority stress theory and social safety perspectives, the research team acknowledged that personal experiences of psychological stress related to being identified as LGBTQ in public would be a potential contributor to a stress response at a highly visible event such as the Gay Games. To account for this possible confounder, past experiences of fear of attending pride events were measured via the following single survey item developed by the research team: “Have you ever felt afraid to go to LGBTQ Pride events?” Response options included “Yes,” “No,” “I don’t know,” and “Prefer not to say.” The responses were dichotomized to represent those who did report past experiences of this fear and those who did not. Those who did not provide a “Yes” or “No” response (n = 2) were treated as missing.

Date of Survey Participation. Our survey item for the outcome variable measured experiences of psychological distress over the past two weeks, aiming for the period of the Gay Games; therefore, it was important to adjust for the date of survey completion during our analysis. The survey launch date occurred at the midpoint of the Gay Games and was coded as 0 = launch date, 1 = next day, etc.

### 2.5. Analysis

Data were analyzed for differences between the three Gay Games participation groups (athlete, spectator/volunteer, and non-attendee). To compare differences by Gay Games participation related to dichotomous or categorical variables, Fisher’s exact test was used to account for some of the small cell sizes (<5); ANOVA was used to compare Gay Games participant groups by continuous variables with the exception of date of survey completion, which utilized a Kruskal–Wallis test due to the non-normality of the data. Multiple imputation (25 imputations) was used to account for missing data in multivariate analyses using PROC MI in SAS to support greater statistical power via sample size. All statistical analyses and imputation were performed using SAS Studio 3.8 on SAS 9.4, and statistical significance was set at *p* < 0.05.

To assess key variables’ unadjusted associations to the outcome variable of interest (psychological distress), bivariate logistic regression analyses were performed. Variables that were significantly associated with the outcome variable in bivariate models—in addition to age, sex assigned at birth, and date of the survey—were included in the first multivariate logistic regression model containing ten variables. Sensitivity analyses were conducted to test the removal of age and sex assigned at birth, as previous research has demonstrated significant associations between these variables and psychological distress [56,57]; similarly we tested the removal of the date of survey participation variable, as some respondents completed the survey during the Gay Games period and some in the immediate period after. These sensitivity analyses yielded no difference in the significance of associations to psychological distress from the 10-variable model to the model removing age, sex assigned at birth, and survey date; characteristics that remained significant in both models (ORs presented for the 10-variable and 7-variable model, respectively) included the following: athletes vs. non-attendees (ORs: 0.297, 0.235), transgender identity (ORs: 4.16, 2.74), fear of pride events (ORs: 3.659, 3.87), and good self-rated health (ORs: 0.106, 0.081).

Then, we sought a most parsimonious model through backward elimination, especially to prevent overfitting a model with a small number of cases (i.e., 40 individuals with a distress score indicating mild distress or more). This process was implemented to maintain a robust events-to-predictors ratio and resulted in the final model including the following variables: Gay Games participation, transgender identity, self-rated health, and fear of pride events. Within each step of the backwards elimination process, we evaluated the AIC statistic to ensure parsimony was not sacrificing model fit; from the 10-variable model to the final four-variable model, AIC remained almost the same, increasing by a value of 0.26 (124.43 to 124.69).

## 3. Results

### 3.1. Demographics

Table 1 displays the demographic characteristics of this sample. Overall, the sample was largely cisgender (83.78%), assigned male at birth (75.68%), and reported at least some university education (81.98%). Nearly 60% of those who reported their income reported earning 7000 Mexican pesos or more biweekly, and approximately half of the sample reported being a member of an LGBTQ association. On average, respondents completed the survey on the third day post-launch. No significant differences were found across Gay Games participation types for any of these aforementioned variables.

Current residence (Mexican state) and age did differ significantly between Gay Games participation groups. Non-attendees predominantly reported living in Jalisco or CDMX, spectators/volunteers overwhelmingly came from the state of Jalisco (the host state), and athletes came from a broader range of states within Mexico; among athletes, over half resided in Jalisco or CDMX. The average age for athletes was slightly older (36) than spectators and volunteers (31) and non-attendees (33).

### 3.2. Self-Reported Health Status, Past Experiences of Discrimination and Fear of Pride Events

There were significant differences among the Gay Games participation types related to the outcome variable of interest in this study: psychological distress (see Table 2); while less than 20% of athletes reported any psychological distress, approximately 36.6% of spectators/volunteers reported distress, as did 50% of non-attendees. The sample reported a high level of self-rated health (only 12.84% reported fair/poor health), despite a substantial minority having been diagnosed with HIV (18.92%). Overall, 90.9% of participants reported at least one instance of public discrimination within their lifetime, 84.7% reported at least one instance of discrimination from family or friends, and 64% reported at least one instance of discrimination in healthcare—without differences across participation type. Additionally, 40.5% had previously feared attending Pride events, with no variation by participation type.

### 3.3. Bivariate Associations with Outcome Variable of Interest (Psychological Distress)

As described above, bivariate analyses (Table 3) were conducted to examine unadjusted associations between psychological distress and several potentially confounding variables that could obscure the relationship between this outcome and Gay Games participation type. These analyses preceded multivariate analyses to determine variables that remained significantly associated with distress. All results of bivariate analyses can be found in Table 3. There is a risk for inflated type I errors from the multiple comparisons within these bivariate analyses; the *p*-values listed in Table 3 should therefore be interpreted descriptively. We elaborate more on the multivariate findings within the results and discussion sections below.

Gay Games Participation. Bivariate analyses demonstrated that Gay Games athletes had lower odds of reporting any psychological distress compared to non-attendees (OR: 0.216; CI: 0.072, 0.646), while spectators/volunteers did not differ significantly from non-attendees.

Sex/Gender. Bivariate analyses demonstrated that reporting a transgender identity was significantly associated with increased odds of distress (OR: 3.299; CI: 1.163, 9.858), whereas sex assigned at birth had no significant association with psychological distress.

Income. Bivariate analyses showed a significant relationship between the dichotomous income variable and psychological distress, with those reporting a low income having 3.585 times the odds (CI: 1.524, 8.432) of reporting psychological distress.

Fear of Pride Events. Bivariate analyses indicated a significant relationship between ever feeling afraid to attend pride events and psychological distress (OR: 3.080, CI: 1.383, 6.858).

Self-Rated Health. Results from bivariate analysis revealed that the dichotomous self-rated health variable significantly predicted psychological distress, with better health associated with lower odds of distress (OR: 0.126; CI: 0.033, 0.485).

Public Discrimination. Bivariate analyses showed a significant relationship between past public discrimination and psychological distress, with those with greater reported discrimination in public settings having 3.921 (CI: 1.032, 14.899) times the odds of reporting psychological distress.

Healthcare Discrimination. Bivariate analyses showed a significant relationship between past healthcare discrimination and psychological distress, with those with greater reported healthcare discrimination having 3.268 (CI: 1.271, 8.403) times the odds of reporting psychological distress.

Other Demographics/Characteristics. Bivariate analyses demonstrated that having received a college/university degree, HIV status, Mexican state of residence, membership within an LGBTQ organization, and age were all not significantly associated with psychological distress in our sample.

Date of Survey Participation. To examine whether the date during which the participants took the survey could have a significant effect on the reported outcome variable, we included this in bivariate analyses, which did not demonstrate a significant association.

### 3.4. Multivariate Associations with Psychological Distress

Results from multivariate analysis are presented in Table 4. The odds ratios and confidence intervals reported for the final model were obtained by pooling results from imputed datasets. In the adjusted model, personal income and past discrimination (which were significant in unadjusted analyses) were not independently associated with distress once variables like self-rated health and fear were included, suggesting those factors may mediate or confound the influence of discrimination and socioeconomic status.

Gay Games Participation (adjusted). With our first and second *a priori* hypotheses (H1 & H2), we expected that athletes would report lower odds of psychological distress than non-attendees and tested this via logistic regression. When adjusting for potential confounders in these multivariate analyses, athletes indeed continued to report significantly lower odds of psychological distress (OR: 0.200; CI: 0.063, 0.630) compared to non-attendees. Our other two *a priori* hypotheses suggested that spectators/volunteers would also report lower odds of psychological distress compared to non-attendees. While spectators/volunteers did not have significantly different odds of psychological distress in bivariate analyses compared to non-attendees, odds of psychological distress were significantly lower than among non-attendees when adjusting for the other variables in the model (OR: 0.310; CI: 0.097, 0.961).

Transgender identity (adjusted). Higher odds of distress among transgender people compared to cisgender people became further exaggerated after adjusting for Gay Games participation profile, self-reported health, and self-reported previous experience of fear during pride events (OR: 4.582; CI: 1.392, 15.071).

Fear of Pride Events (adjusted). Multivariate analyses continued to indicate a significant positive association between ever feeling afraid to attend pride events and psychological distress (OR: 4.583; CI: 1.692, 12.401) after adjusting for Gay Games participation type, transgender identity, and self-reported health.

Self-Rated Health (adjusted). After adjusting for Gay Games participation type, transgender identity, and self-reported previous experience of fear during pride events, those who reported their health to be good, very good, or excellent had 0.101 (0.014, 0.708) times the odds of reporting any psychological distress compared to those reporting their health as fair or poor.

## 4. Discussion

This study suggests a positive relationship between Gay Games participation and mental health within our sample of LGBTQ Mexican residents. The multivariate results presented in Table 4 demonstrate that after adjusting for potential confounders, LGBTQ people participating in the event—particularly those who participated as athletes—exhibited a lower likelihood of psychological distress than their counterparts who did not attend the Gay Games. While our study design cannot confirm causality due to this study’s cross-sectional design, this study is the first to document mental health correlates of Gay Games participation. These results that lower distress among participants, especially athletes, are consistent with that sports participation and physical activity are connected to mental health [16,17,19,20,21], possibly via a sense of belonging [58], achievement [59], and community support [60]. Individuals from marginalized groups may lack substantial social safety in of daily life; therefore, the presence of thousands of other openly LGBTQ athletes and supporters has the potential to boost positive affect and reduce stress, even if only in the short term. This study extends the existing evidence base into an LGBTQ-focused sporting context. Results from our multivariate analyses also suggest a potential difference in the odds of reported psychological distress between spectators/volunteers and non-attendees; however, the large relative standard errors from the small group sizes indicate an inability to claim with certainty the statistical significance regarding a difference between these two groups. Future investigation with a larger sample size would add valuable insight into the robustness of this relationship, and the possibility of a dose–response effect of Gay Games participation. For example, are all those who are present at the event experiencing lower distress via the feelings of social safety and connection to community? Is there an added benefit to those who engage in competition via physical activity? Or, as previously explored in the context of the Gay Games [3], social identity theory’s explanation of the role of identity affirmation in boosting self–esteem and belonging could lend insight to why all attendees—spectators/volunteers and athletes—might experience greater odds of mental wellness; indeed, this could also provide potential rationale for athletes’ more robust connection to positive mental health, as their community connection could be enhanced via competing alongside teammates towards a common goal.

A striking aspect of our results is the magnitude of the difference in distress prevalence in our sample of athletes and non-attendees (19% vs. 50%, respectively). This suggests the potential for a substantially impactful mechanism for mental health promotion, if indeed there is a causal association between Gay Games participation and distress.

Despite not being able to ascertain the causal rationale related to the four hypotheses stated above, this study points to some next steps for future research on this topic. Our results supports the rejection of the null hypotheses of H1, H2, H3 and H4, as these each state that there would be no significant differences in relative likelihood of lower psychological distress between different Gay Games participation groups; in particular, the robustness of the finding that athletes reported significantly lower odds of psychological distress compared to non-attendees is compelling rationale for further testing of H1 and H2 with study designs that can strengthen confidence in causal pathways. Additional future research with a larger sample that tests whether greater Gay Games involvement (participating vs. spectating vs. non-attending) corresponds with progressively better mental health is necessary to enhance confidence in H3 and H4 in lending insight to specific aspects of the Gay Games that support mental well-being, including whether simply being in an affirming environment is sufficient for improving mental health, or if participating in a sport (and thereby benefitting from physical activity and team camaraderie) is necessary for a mental health benefit. As stated previously, even sitting among others in the stands celebrating LGBTQ visibility in sports may offer sufficient social safety to benefit mental health, even if one is not experiencing such benefits via physical activity at the event.

While our study design did not provide the opportunity to assess baseline mental health at a timepoint prior to the Gay Games, Table 2 demonstrates the relative similarity between participation groups across several demographic characteristics and indicators of health status and discrimination. While the characteristics overall varied very little across participation groups, they are worth noting; the athlete group was on average between four and five years older than the other participation types denoted in our sample and was more geographically diverse in terms of Mexican state of residence. Because age is often related to psychological distress [61], we included this variable in our initial adjusted models. Multivariate analyses suggested that age in and of itself was not predictive of psychological distress. Anecdotally, we understand that the average age of athletes in our sample was generally reflected in trends of the average Gay Games athlete’s age, both historically and at this event in Mexico. Therefore, investigating barriers to younger LGBTQ people’s participation at the Gay Games could lend insight to other factors that account for differences in mental health among Gay Games athletes. Similarly, study of factors related to the geographic diversity from which Gay Games athletes in our sample could also elucidate missing elements of the causal pathway.

While we recommend exploring the variables referenced above, the similarities in potential risk factors for distress among the participation group used for analysis (i.e., self-rated health, HIV status, fear of Pride events and experiences of discrimination) suggest that how groups participated in the Gay Games was the primary factor measured within this study that differentiated them. While we have insufficient rationale to claim causality due to the relative between-group similarity that could be a feature of our sampling strategy, this is an important exploratory finding that can guide future research and practice at the intersections of mental health and inclusive sports. If other investigations of this topic provide further support for an association between Gay Games participation and mental well-being, a strategy to improve LGBTQ mental health could be implemented and tested, regardless of which of our following hypothesized causal pathways is most plausible: (1) inclusive sporting events themselves positively influence mental health among LGBTQ people; or (2) more mentally well LGBTQ people participate in or attend inclusive sporting events. The organizers of LGBTQ-inclusive events such as the Gay Games, therefore, should continue to address barriers to participation from all parts of the LGBTQ community, particularly those that are most at risk of experiencing poor mental health; extensive outreach to those most lacking opportunities for social inclusion and belonging—including those who may already experience poorer physical or mental health than the traditional Gay Games audience—may provide an opportunity to further manifest the vision of this event. Future evaluation of such outreach efforts and barriers to participation may further lend support for or against our stated hypotheses and clarify additional action steps to continue to promote mental health among LGBTQ individuals in various settings.

### 4.1. Transgender Individuals and Mental Health

Our results highlight specific mental health concerns for transgender individuals, even within LGBTQ-affirming spaces. When holding Gay Games participation and other variables constant, transgender people exhibited over four times the odds of reporting psychological distress compared to cisgender people, reflecting their heightened vulnerability to mental health challenges, likely exacerbated by systemic discrimination, social exclusion, and limited access to affirming healthcare [62]. Elevated distress among this group is perhaps unsurprising given the currently hostile rhetoric about transgender people in sports [63,64]. The significance and magnitude of the increased odds of distress also could be expected in the context of our hypotheses about social safety; as transgender participation is relatively low in comparison to the largely cisgender (male) attendance of the Gay Games, there may be an insufficient visibility or density—whether actual or perceived—of other transgender people with whom to feel safe. It is important that future evaluations of the Gay Games assess how well the event supports the specific needs of gender-expansive individuals, to help reduce distress during the event and provide a buffer against the hostility they may face outside of it. The current lack of accessible, inclusive sports spaces is most often the biggest barrier to transgender people participating in athletics [65], accentuating not only the opportunity for, but also the obligation of, the Gay Games to utilize its international visibility to engage greater numbers of transgender people in safe, affirming, and friendly competition.

Another key finding is the high proportion of participants (40.5%) reporting that they had ever felt fear during pride events (even among athletes, 34.4% reported fear). This may reflect broader social stigmas and safety concerns that persist even within LGBTQ spaces and events [66], as anti-LGBTQ activities have increased at Pride events in the context of hostile social rhetoric [67]. Moreover, our analysis showed that fear during attending pride events was a significant predictor of psychological distress when adjusting for potential confounders, whereas variables representing past experiences of discrimination in healthcare settings or within the general public were not consistently significant after such adjustment. Since our bivariate analyses indeed demonstrate that past discrimination within public and healthcare settings is associated with distress, it poses questions about whether the discriminatory events themselves contribute directly to ongoing distress, or the internalized fear and perceived threat associated with being visibly LGBTQ in spaces like Pride is more influential. This distinction between the hostility people have experienced and the hostility they fear is yet to occur highlights an important nuance in understanding LGBTQ experiences; as elevated hostility within the social environments of LGBTQ people is associated with severe mental health outcomes (e.g., suicide attempts) [41], it is imperative to establish and maintain genuinely affirming spaces that can foster a sense of safety and support for mental well-being. This study suggests that the Gay Games has the potential to be such a space. Further research in this area could lend insight to how to reduce fear or unease among Pride attendees of this or other LGBTQ events, perhaps through interventions to process and neutralize fear responses for those wanted to gather in queer community. This finding could inspire future Gay Games organizers to include additional social safety efforts—informed by consultation with transgender athletes and community leaders themselves—to ensure that benefits of the event are felt over and above any fear contributing to distress.

### 4.2. LGBTQ Association Membership and Mental Health

Within the present analysis, being a member of an LGBTQ organization was not associated with lower psychological distress. This contrasts with findings that generally show a positive association between participation in LGBTQ spaces and mental health [68,69]. It is possible that adjusting for the present environment of the Gay Games, which may promote mental well-being even if an individual does not generally engage within LGBTQ associations, may have contributed to a null association. Alternatively, it may be possible that for many Mexicans the stressors associated with being LGBTQ outweighs the protective factors of engagement with LGBTQ community. More research within this specific population is necessary to elucidate how LGBTQ associations within Mexico can continue to promote mental wellness.

Another possible explanation for the lack of significant association could be that reported association membership is an imperfect proxy for social support. Possible factors that could obscure true associations could include those who do not consider themselves members of a formal association but still have substantial social support, as well as those who are members that might experience stressors through internal community conflict or stress related to time commitment or obligation. More refined measures that distinguish social support from community participation through formal membership are warranted.

### 4.3. HIV Status and Mental Health

Finally, it is notable that our sample featured a relatively high proportion of persons having been diagnosed with HIV compared to the overall LGBTQ population in Mexico. The most recent data listed by UNAIDS at the time of this publication estimates the HIV prevalence among men who have sex with men (MSM) in Mexico to be 11.9% [70]. The prevalence within our sample (18.9%) may be higher than in the country overall because of recruitment strategies used in our study that featured outreach to HIV prevention and service organizations that worked with LGBTQ communities. These strategies, while effective in reaching LGBTQ persons in different locations within Mexico, likely result in an oversampling of persons living with HIV. Indeed, others studying HIV among Mexican MSM who have implemented recruitment strategies within higher-risk settings have estimated the prevalence to be approximately 17% [71]. While our sample is not limited to MSM, a vast majority of our sample identified as men within the LGBTQ community; therefore, the similarity in HIV prevalence between our sample is comparable to others with similar methods within this population. Notably, self-reported past HIV diagnosis was not associated with psychological distress in our sample (Table 3); therefore, the overrepresentation of people living with HIV likely did not bias our findings related to Gay Games participation and mental health.

### 4.4. Potential for Real-World Application

Our findings suggest aspects of sporting events which may be modified to promote mental health for LGBTQ participants. Examples of potential strategies are presented below; future research that can better assess causality should investigate further the possible impacts of these or similar approaches for the diverse identities represented within the LGBTQ community.

Recommendation 1—Proactive Mitigation of Safety Concerns. Our findings suggest that LGBTQ individuals’ experiences of fear at pride events may at least somewhat relate to any benefits experienced at events like the Gay Games. Addressing potential participants’ safety concerns and making clear the strategies being implemented to address them prior to and during the event could extend potential benefits of the Gay Games to a greater number of individuals.

Recommendation 2—Support for Transgender Individuals. Our results suggest that transgender and gender diverse people may differently experience the Gay Games’ effects on mental health. Ensuring that mental health supports specific to transgender people are available at such events may better support the actualization of any mental health benefit from their participation. Such resources may include interventions designed to address mental health concerns (i.e., crisis response, gender-affirming mental health campaigns or services) as well as providing intentional space for transgender people to experience sport in an environment of social safety (i.e., gender-inclusive competition/award categories, transgender-specific teams, enforced policies related to anti-discrimination).

Recommendation 3—Support for Persons Living with HIV/AIDS (PLWH). While not a primary objective of the study, our results suggest that events such as the Gay Games may be of interest and accessible to PLWH within in the LGBTQ community. Ensuring that resources are available to support the well-being of participants who are living with HIV, as well as the prevention of HIV transmission during the event, should therefore be considered by event organizers. For example, access to pre-exposure prophylaxis (PrEP) and post-exposure prophylaxis (PEP), would further promote the potential well-being of participants beyond that obtained via physical activity and social safety/community connection.

## 5. Limitations

This study is not without limitations. First, as mentioned above, spatially and/or temporally different sports contexts may consist of dramatically different expressions or homophobia and/or transphobia; this limits the generalizability of these results to other competitions, even those of similar size and focus. Rather than claiming universal generalizability, this study provides another piece of the puzzle in understanding mental health among LGBTQ athletes in an ever-changing landscape. To better understand the role of sports in mental health promotion, future studies could utilize partnerships with LGBTQ organizations to establish evidence related to causality through evaluation designs that minimize possible bias.

Additional limitations relate to the sampling and recruitment strategy. This study utilized a convenience sample, which presents the possibility of selection bias. While athletes and spectators/volunteers could be invited in-person and through social media, non-attendees were only reached through social media of LGBTQ associations. LGBTQ people with less connection to the larger LGBTQ community may have been less likely to learn about the study as a result of our strategy; as less-connected individuals could possibly have different experience of and relief from identity-related stressors, ensuring that even the most vulnerable and isolated LGBTQ individuals have opportunities to learn about future studies is necessary. However, as we did not find any significant difference in membership of an LGBTQ association among the three groups, we believe the bias is minimal. Future study that includes randomized sampling could provide more clarity in the relationship between LGBTQ-affirming participation and psychological distress.

The size of the sample in this study also presents limitations in the conclusions that can be made from our analyses, as some analyses may have been underpowered in detecting small differences. Sample size relative to the sampling frame is one such possible limitation; with over 2500 athletes registered for the Gay Games, our sample is relatively small. Gay Games reporting has stated that approximately 25% of registered athletes were from Mexico, suggesting that there were 600–750 Mexican athletes alone eligible to participate in the survey. While the sample size is rather small, the relative similarity of the demographics to the Gay Games athlete population provides support for our results’ generalizability to the larger population. Additionally, the size of the participation groups—especially the spectator/volunteer group—poses limitations to the certainty that can be applied to conclusions about between-group differences.

In addition to the limitations of any cross-sectional study, other limitations relate to the survey measures used to evaluate mental health. We used a relatively low cutoff point (a score of 3 on the PHQ-4 measure) to indicate any psychological distress. As it is unclear the extent to which there would be clinical significance for lower levels of distress, a higher cutoff point or continuous measure could be explored to further understanding of differences in associations to greater, and possibly more clinically relevant, levels. However, this cutoff point has been used in other instances to assess any amount of psychological distress, even if such reported levels are not warranting immediate mental health treatment for acute crises. Additionally, we cannot rule out the possibility that unmeasured variables (such as resilience or history of mental health conditions) might have influenced a person’s likelihood of participating in the Gay Games that also account for part of one’s mental wellness.

Our study’s exploration of Gay Games participation in relation to mental well-being is a starting point for assessing potential benefits yet remains limited in its ability to suggest what specific factors of such inclusive sporting events are most impactful. The present analyses did not account for athletic activity independent from Gay Games participation; for example, in the surrounding time frame, Gay Games participants may have engaged in sports in addition to ones they competed in during the event, and others not competing in Gay Games may have indeed been athletes themselves with no intention or ability to compete at this prominent event. The Gay Games’ status as an amateur event was indeed open to all, but costs associated with registration or travel, ability to take time away from work or family obligations, and comfort with visible affiliation with an LGBTQ event could obscure the true association of our variables of interest if many spectators/volunteers or non-attendees are indeed athletes in other settings.

Even though this study was set within the context of an international event, we are unable to generalize too widely to different contexts or populations other than those investigated here. First, this study analyzed a limited sample of residents participating in an international sporting event hosted within their home country; therefore, just as we are limited in our ability to generalize the association between participation and mental health to those of other nationalities competing in the 2023 Gay Games, we are also limited in our ability to generalize these results to LGBTQ people of other nationalities.

Finally, the cross-sectional nature of this data precludes our ability to determine causality; whether the Gay Games promote positive mental health or simply attract a more mentally well population is unable to be determined with this study design. Despite this limitation, our results can still inform Gay Games and other sporting event organizers in considering the mental health needs of LGBTQ athlete populations and of the communities within which their events take place.

## 6. Conclusions

The Gay Games and other LGBTQ events—sporting or otherwise—provide important opportunities for sexual and gender diverse people to connect, play, and grow in community, potentially buffered from the hostile sociopolitical environments in many parts of the world in the present day. The results of our analysis suggest that people most engaged in the Gay Games—the athletes competing in competition—may exhibit better mental health than those not participating at all. Our findings lay the groundwork for future research to test whether and how similar events can promote mental health. Studies that can better assess causality through longitudinal or experimental approaches would justify the implementation of strategies that may best produce such outcomes for the athletes, other attendees, and the broader community which these events aim to serve. We have demonstrated that sport can indeed be a place where underrepresented athletes can thrive, especially when threats from the outside world may be limited. The Gay Games, therefore, may not be a microcosm of the world today, but of the world that we can create by fostering respect, inclusion, and affirmation of LGBTQ communities in realms beyond and within sport itself.

## Figures and Tables

**Table 1:** Demographic characteristics by Gay Games Participation Type (n = 111).

Variable	Overall n (%)	Athlete n (%)	Spec/Vol n (%)	Non-Attendee n (%)	Fisher’s Exact *p* *
	111 (100.00)	32 (28.83)	41 (36.94)	38 (34.23)	
Age, mean (SD)					0.0167 ***^+^***
	33.10 (6.87)	35.83 (5.07)	31.02 (6.44)	33.11 (7.92)	
Sex at Birth, n (%)					0.3258
Female	27 (24.32)	9 (28.13)	12 (29.27)	6 (15.79)	
Male	84 (75.68)	23 (71.88)	29 (70.73)	32 (84.21)	
Transgender, n (%)					0.1728
Yes	18 (16.22)	2 (6.25)	9 (21.95)	7 (18.42)	
No	93 (83.78)	30 (93.75)	32 (78.05)	31 (81.58)	
Education, n (%)					0.2289
Some University	91 (81.98)	29 (90.63)	34 (82.93)	28 (73.68)	
No University	13 (11.71)	1 (3.13)	4 (9.76)	8 (21.605	
Missing	7 (6.31)	2 (6.25)	3 (7.32)	2 (5.26)	
Income, n (%)					0.0792
<7K biweekly	40 (36.04)	6 (18.75)	17 (41.46)	17 (44.74)	
7K+ biweekly	59 (53.15)	21 (65.63)	22 (53.66)	16 (42.11)	
Missing	12 (10.81)	5 (15.63)	2 (4.88)	5 (13.16)	
Residence (Mexican State), n (%)					<0.0001 *
Jalisco	54 (48.65)	12 (37.50)	32 (78.05)	10 (26.32)	
CDMX	35 (31.53)	10 (31.25)	5 (12.20)	20 (52.63)	
(State of) Mexico	6 (5.41)	2 (6.25)	0 (0.00)	4 (10.53)	
Other	11 (9.91)	7 (21.88)	2 (4.88)	2 (5.26)	
Missing	5 (4.50)	1 (3.13)	2 (4.88)	2 (5.26)	
LGBTQ Association, n (%)					0.8511
Member	49 (44.14)	16 (50.00)	19 (46.34)	14 (36.84)	
Non-Member	59 (53.15)	15 (46.88)	21 (51.22)	23 (60.53)	
Missing	3 (2.70)	1 (3.13)	1 (2.44)	1 (2.63)	
Date of Survey, mean (SD)					0.1171 †
	2.67 (2.97)	2.21 (3.10)	3.20 (3.21)	2.53 (2.44)	

* Significance: *p* < 0.05. ^+^ ANOVA test. † Kruskal–Wallis test.

**Table 2:** Self-Reported Health, Experiences of Discrimination and Fear of Pride Events by Gay Games Participation Type (n = 111).

Variable	Overall n (%)	Athlete n (%)	Spec/Vol n (%)	Non-Attendee n (%)	Fisher’s Exact *p* *
	111 (100.00)	32 (28.83)	41 (36.94)	38 (34.23)	
Any Psychological Distress, n (%)					0.0255 *
Yes	40 (36.04)	6 (18.75)	15 (36.59)	19 (50.00)	
No	71 (63.96)	26 (81.25)	26 (63.41)	19 (50.00)	
Self-Rated Health, n (%)					0.8218
Excellent/Good	95 (87.16)	29 (90.63)	35 (85.37)	31 (86.11)	
Fair/Poor	14 (12.84)	3 (9.38)	6 (14.63)	5 (13.89)	
Missing	2 (1.80)	0 (0.00)	0 (0.00)	2 (5.36)	
HIV+ Diagnosis, n (%)					0.5339
Yes	21 (18.92)	4 (12.50)	11 (26.83)	6 (15.79)	
No	85 (76.58)	26 (81.25)	29 (70.73)	30 (78.95)	
Missing	5 (4.50)	2 (6.25)	1 (2.44)	2 (5.26)	
Fear of Pride Events, n (%)					0.5515
Yes	45 (40.54)	11 (34.38)	20 (48.78)	14 (36.84)	
No	64 (57.66)	20 (62.50)	21 (51.22)	23 (60.53)	
Missing	2 (1.80)	1 (3.13)	0 (0.00)	1 (2.63)	
Public Discrimination, mean (SD)					0.2360 **^+^**
	0.498 (0.30)	0.457 (0.30)	0.562 (0.27)	0.464 (0.33)	
Healthcare Discrimination, mean (SD)					0.2664 **^+^**
	0.513 (0.43)	0.438 (0.44)	0.598 (0.42)	0.487 (0.44)	
Family & Friends Discrimination, mean (SD)					0.2505 **^+^**
	0.684 (0.37)	0.615 (0.38)	0.756 (0.34)	0.667 (0.39)	

* *p* value significant at 0.05. ^+^ ANOVA test.

**Table 3:** Unadjusted Odds of Reporting Any Psychological Distress among a Sample of LGBTQ Mexicans During the 2023 Gay Games (n = 111).

Variable	No Distress n (%)	Distress n (%)	Unadj OR (95% CI) ^+^
	71 (63.96)	40 (36.04)	
Participation Type, n (%)			
Athlete	26 (81.25)	6 (18.75)	0.216 (0.072, 0.646)
Spectator/Volunteer	26 (63.41)	15 (36.59)	0.500 (0.294, 1.226)
Non-Attendee	19 (50.00)	19 (50.00)	−ref
Sex at Birth, n (%)			
Female	21 (77.78)	6 (22.22)	0.400 (0.146, 1.093)
Male	50 (59.52)	34 (40.48)	−ref
Transgender, n (%)			
Yes	7 (38.89)	11 (61.11)	3.299 (1.163, 9.858)
No	64 (68.82)	29 (31.18)	−ref
Education, n (%)			
University	58 (65.17)	31 (34.83)	0.633 (0.196, 2.043)
No University	7 (53.85)	6 (46.15)	−ref
Income, n (%)			
<7K biweekly	18 (45.00)	22 (55.00)	3.585 (1.524, 8.432)
7k+ biweekly	43 (75.44)	14 (24.56)	−ref
Mexican State, n (%)			
CDMX	18 (54.55)	15 (45.45)	1.623 (0.673, 3.915)
Mexico	4 (66.67)	2 (33.33)	0.974 (0.163, 5.804)
Other	8 (72.73)	3 (27.27)	0.730 (0.173, 3.075)
Jalisco	36 (66.67)	18 (33.33)	−ref
LGBTQ Association, n (%)			
Member	39 (66.10)	20 (40.82)	1.248 (0.572, 2.723)
Non-Member	29 (59.18)	20 (33.90)	−ref
Fear of Pride Events, n (%)			
Yes	21 (46.67)	24 (53.33)	3.080 (1.383, 6.858)
No	48 (75.00)	16 (25.00)	−ref
Self-Rated Health, n (%)			
Excellent/Good	64 (68.82)	29 (31.18)	0.126 (0.033, 0.485)
Poor/Fair	3 (21.43)	11 (78.57)	−ref
Diagnosed w/HIV, n (%)			
Yes	12 (60.00)	8 (40.00)	1.125 (0.416, 3.045)
No	53 (63.10)	31 (36.90)	−ref
Age, mean (SD)			
	34.15 (7.0)	31.63 (6.49)	0.946 (0.892, 1.004)
Public Discrimination, mean (SD)			
	0.455 (0.30)	0.576 (0.30)	3.921 (1.032, 14.899)
Healthcare Discrimination, mean (SD)			
	0.437 (0.43)	0.650 (0.41)	3.268 (1.271, 8.403)
Family/Friends Discrimination, mean (SD)			
	0.634 (0.38)	0.775 (0.32)	3.095 (0.973, 9.846)
Date of Survey, mean (SD)			
	2.761 (3.12)	2.500 (2.73)	0.970 (0.848, 1.109)

^+^ Significance: *p* < 0.05.

**Table 4:** Final Model with Adjusted Odds of Reporting Any Psychological Distress among a Sample of LGBTQ Mexicans During the 2023 Gay Games (n = 111).

Variable	No Distress n (%)	Distress n (%)	Unadj OR (95% CI)	Adj OR (95% CI) ^+^
Participation Type, n (%)				
Athlete	26 (81.25)	6 (18.75)	0.216 (0.072, 0.646)	0.200 (0.063, 0.630)
Spectator/Volunteer	26 (63.41)	15 (36.59)	0.500 (0.294, 1.226)	0.310 (0.097, 0.961)
Non-Attendee	19 (50.00)	19 (50.00)	−ref	−ref
Transgender, n (%)				
Yes	7 (38.89)	11 (61.11)	3.299 (1.163, 9.858)	4.582 (1.392, 15.071)
No	64 (68.82)	29 (31.18)	−ref	−ref
Fear of Pride Events, n (%)				
Yes	21 (46.67)	24 (53.33)	3.080 (1.383, 6.858)	4.583 (1.692, 12.401)
No	48 (75.00)	16 (25.00)	−ref	−ref
Self-Rated Health, n (%)				
Excellent/Good	64 (68.82)	29 (31.18)	0.126 (0.033, 0.485)	0.101 (0.014, 0.708)
Poor/Fair	3 (21.43)	11 (78.57)	−ref	−ref

^+^ Significance: *p* < 0.05.

## Data Availability

The datasets presented in this article are not readily available because the IRB-approved condition of consent provided by human subjects assured that their information would not be distributed or shared for future studies without their expressed permission.
